# Delta-wave automatic mapping of the manifest accessory pathway

**DOI:** 10.3389/fcvm.2024.1449038

**Published:** 2024-08-21

**Authors:** Saverio Iacopino, Gennaro Fabiano, Paolo Sorrenti, Andrea Petretta, Jacopo Colella, Alessandro Di Vilio, Giovanni Statuto, Nicolangelo Diomede, Paolo Artale, Pasquale Filannino, Antonino Pardeo, Filippo Placentino, Giuseppe Campagna, Gianluca Peluso, Edoardo Cecchini, Federico Cecchini, Giuseppe Speziale, Fiorenzo Gaita

**Affiliations:** ^1^ Electrophysiology Department, Maria Cecilia Hospital, GVM Care & Research, Cotignola, Italy; ^2^ Electrophysiology Department, Anthea Hospital, GVM Care & Research, Bari, Italy; ^3^ Electrophysiology Department, San Carlo di Nancy, GVM Care & Research, Roma, Italy

**Keywords:** Wolff-Parkinson-White Syndrome, accessory pathway, 3D electro-anatomical mapping system, activation map, automatic signal annotation, radiofrequency catheter ablation

## Abstract

**Background:**

Despite the high success rate of radiofrequency catheter ablation (RFCA) in Wolff-Parkinson-White Syndrome (WPW), localizing the successful ablation site can be challenging and may require multiple radiofrequency (RF) applications.

**Objective:**

This study aims to evaluate the efficacy of a novel workflow for the automatic and precise identification of accessory pathway ablation site, named Delta Wave Automatic Mapping.

**Methods:**

Patients undergoing a first procedure for RF ablation of a manifest accessory pathway were included. Electro-Anatomical Mapping (EAM) was performed with the CARTO 3 system (Biosense Webster, Johnson & Johnson Medical S.p.a., Irvine, CA) leveraging auto-acquisition algorithms already present in the CARTO 3 software. Mapping and ablation were performed with an irrigated tip catheter with contact force sensor. Procedure success was defined as loss of pathway function after ablation. The number of RF applications required and time to effect were measured for each patient. Recurrences were evaluated during follow-up visits. Additionally, at the end of each procedure, historical predictors of ablation success were measured offline to evaluate their relationship with the successful ablation site found with the novel workflow.

**Results:**

A total of 50 patients were analysed (62% APs right and 38% APs left). All 50 APs were successfully eliminated in each procedure with a median Time-to-effect (TTE) of 2.0 (IQR 1.2–3.5) seconds. No AP recurrences during a median follow-up of 10 (IQR 6–13) months were recorded. Offline analysis of successful ablation site revealed a pre-ablation delta/ventricular interval of ≤−10 msec in 52% of the patients and in 100% of the patients the signal related to the Kent bundle was identified.

**Conclusions:**

The novel workflow efficiently localizes APs without requiring manual annotations. Historical endocardial parameters predicting success were measured offline for each case and they corresponded to the ablation target automatically annotated by the proposed workflow. This novel mapping workflow holds promise in enhancing the efficacy of RFCA in the presence of manifest APs.

## 1 Introduction

According to the current guidelines (1) catheter ablation of AP is recommended either for patients with a clinical history of AVRT, either in asymptomatic patients with a high-risk profile for specific electrophysiological (EP) parameters.Despite the safety and the high success rate of catheter ablation (ranging from 90% for right sided pathways, up to 97% for left sided pathways) there is still a non-negligible rate of failure (3%−10%) (2–4).

Catheter technology, combined with 3D navigation systems that enable real-time anatomy reconstruction and electro-anatomical (EA) data acquisition, may help in optimizing AP procedures (5). However, conventionally, even when using EAM, AP signal annotation is performed manually with the clinical specialist setting the caliper of the time annotation on the earliest second signal found. This process is easily prone to error and imprecision. Open Window Mapping (OWM) techniques offer advantages over conventional methods by automatically detecting local signals and eliminating the need for manual EGM identification. OWM requires to set a window of interest (WOI) that encloses both the atrial and ventricular signals. Moreover, OWM requires the use of a multipolar catheter to achieve high density maps around the annulus where the the system annotates the earliest signal found (without distinction between atrial and ventricular component). Afterwords, additional features, such as the extended early meets late (EEML) of the Carto 3® software, will then process the signals and delineate a white line around the annulus with an interruption or gap corresponding to the area where the system did not detect a sufficiently long time interval between the atrial and ventricular component, hence indicating the AP location (6).

The automatic annotation with OWM solves the problem of manually setting the time caliper, however it requires the use of multipolar mapping catheters to achieve highly dense maps which are key elements for proper EEML and gap visualisation. Furthermore, OWM requires to manually adjust the EEML threshold to visualize the gap (7).

With the aim of achieving automatic, fast and accurate manifest AP localization minimizing the total number of catheters used, we have developed a multi-parameter technique based on EA mapping using the CARTO® 3 system (Biosense Webster, Johnson & Johnson Medical S.p.a., Irvine, CA) and we called it Delta Wave Automatic Mapping.

## 2 Materials and methods

### 2.1 Study population

The study consisted of 50 consecutive patients who underwent an electrophysiological study (EPS) and radiofrequency catheter ablation (RFCA) of manifest AP using the CARTO® 3 electroanatomic mapping system, at two institutions, between June 2019 and September 2023. The study protocol received approval by the hospitals’ institutional review board and complied with the declaration of Helsinki.

The only inclusion criteria was the presence of a manifest AP whereas exclusion criteria were previous RFCA of AP and presence of congenital heart disease.

### 2.2 Electrophysiology study

Antiarrhythmic drugs were discontinued at least 5 days prior the procedure.

Surface ECG, bipolar and unipolar signals were continuously recorded and stored on the WorkMate™ Claris™EP stimulator (St. Jude Medical, Minneapolis, MN, USA).

Procedures were performed under local anesthesia and conscious sedation.

By using the Seldinger's technique, three ultrasound guided vascular accesses of the right femoral vein were obtained. The femoral arterial access was obtained for the retrograde approach to the left APs.

When possible, advancement and placement of the catheters together with mapping and ablation were performed with the intent to avoid x-ray exposure or at least to minimize it.

One standard quadripolar catheter was placed in the right ventricular apex or right ventricular outflow tract and a deflectable bidirectional decapolar catheter was advanced into the coronary sinus (CS). An EPS was carried out to define the electrical properties of the AP.

A ThermoCool or ThermoCool SF SmartTouch ablation catheter (Biosense Webster, Johnson & Johnson Medical S.p.a., Irvine, CA) with contact-force sensor was used for mapping and ablation.

If the AP was located around the mitral annulus, choice between trans-septal or retro-aortic approach was left to the physician.

### 2.3 Workflow

We have described the novel workflow for automatic mapping of manifest accessory pathways (AP) with CARTO® 3 system in a previous publication (8).

The workflow is based on the concomitant use of the following modules integrated in the CARTO Prime^TM^ software of the CARTO® 3 system, version 7 (Biosense Webster): Confidense^TM^ module, Automatic Mapping, Wavefront Annotation, and Pattern Matching.

First, we acquired the basal ECG signal for Pattern Matching (beat-to-beat analysis on the 12-lead ECG derivation), setting the reference on the most stable QRS peak in one of the 12 ECG leads.

To be as precise as possible, we set specific filters in the Confidense Module for automatic mapping: (a) pattern-matching should correlate at least 96% with the pre-excited QRS morphology (during Sinus rhythm, atrial pacing or antidromic tachycardia); (b) the mapping catheter should be stable (position stability set to 5 mm, LAT stability set to 5 msec); (c) the mapping catheter should have a contact force ≥3 grams.

In our map, information on the local activation time of the manifest AP was acquired, excluding the atrial signals from the WOI; in this way, we always had only the ventricular signal in the reference window. Specifically, the WOI was set from the end of the *P* wave to the end of the QRS complex evaluated on all the 12 ECG leads in sinus rhythm.

**Figure 1:**
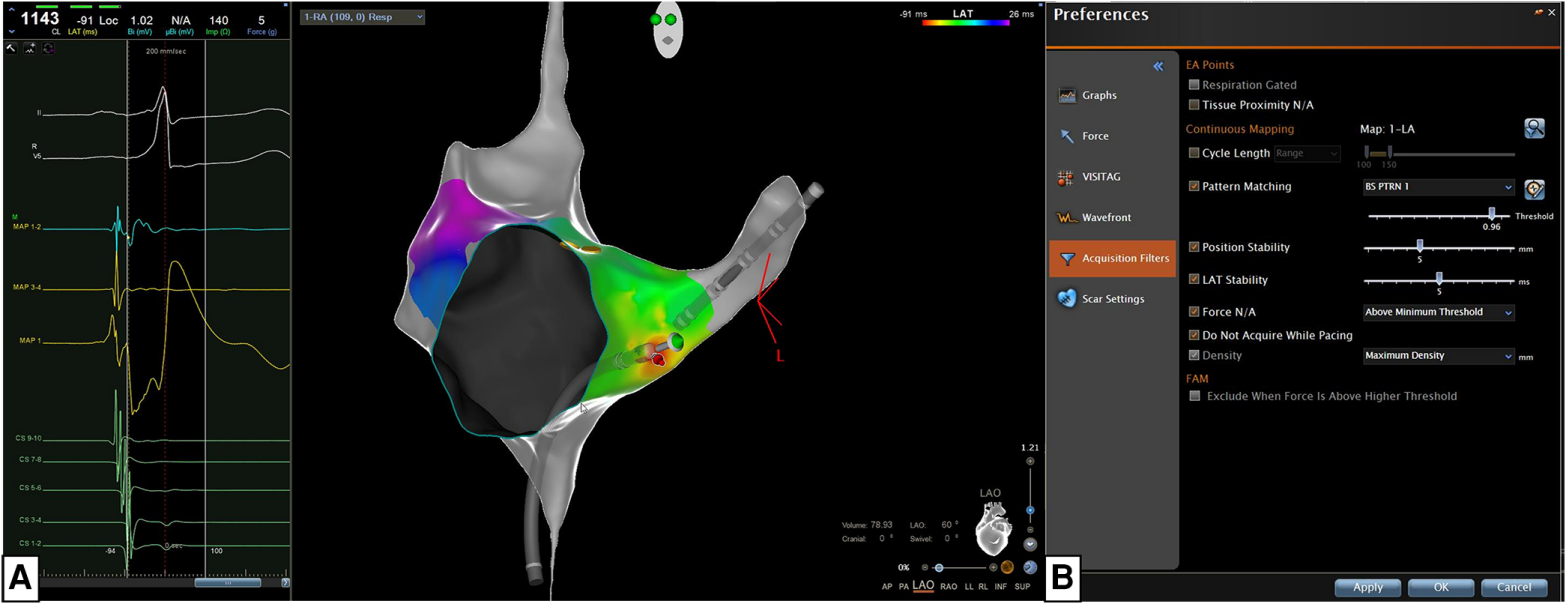
The new algorithm is based on the concomitant use of the confidense^TM^ module (automatic mapping, wavefront annotation, and pattern matching) and the CARTO prime^TM^ parallel mapping module integrated in the CARTO prime^TM^ software of the CARTO 3 system version 7 (biosense webster, Johnson & Johnson medical S.p.a., Irvine, CA). **(A)** The most stable QRS peak in one of the 12 ECG leads was chosen as reference for Pattern Matching (beat-to-beat analysis). **(B)** We set specific filters for the Confidense Module and these filters are the following: (a) pattern-matching should correlate at least 96% with the pre-excited QRS morphology (during Sinus rhythm, atrial pacing or antidromic tachycardia); (b) the mapping catheter should be stable in position and temporal activation (5 mm, 5 s); (c) the mapping catheter should have a contact force ≥3 grams with the endocardial tissue; (d) acquisition density should be set at the maximum level (<1 mm).

The annotation of the ventricular signal in the WOI is based on the Wavefront Annotation algorithm of the CARTO 3 system. This algorithm focuses on the annotation of the maximum negative slope of the unipolar EGM within the WOI defined previously. These settings allow us to automatically annotate the earliest activation signal without any manual adjustment (Figure 1).

In case of intermittent ventricular pre-excitation, atrial stimulation was performed at a cycle length of 500–600 msec from the bipoles of the decapolar catheter placed in the CS, to reach a stable pre-excitation allowing a faster mapping. In this case, the reference was set on the stimulating bipole and the WOI was created to exclude the atrial stimulation spike and the atrial signal.

### 2.4 Radiofrequency catheter ablation

Ablation was systematically performed in the area automatically identified by the mapping system with a ThermoCool or ThermoCool SF SmartTouch ablation catheter (Biosense Webster, Johnson & Johnson Medical S.p.a., Irvine, CA), a power limit of 35–40 W, a contact force between 5 and 25 g, until reaching an ablation index (AI) of 550.

Acute success was defined as the vanishing of AP electrical signals (split of bipolar atrio-ventricular EGM and delta-wave disappearance) during RF application, while the time to effect (TTE) was considered as the time interval between the beginning of RF delivery and the disappearance of AP. After a successful RF application, a waiting period of 30 min was observed. Ventricular and atrial stimulation protocols plus adenosine administration were performed to confirm the absence of conduction resumption over the AP (9). In case of conduction recurrence, ablation was performed after re-mapping the area.

If the AP was not eliminated within 10 s or the catheter moved during the application, RF delivery was interrupted.

### 2.5 Signal analysis

The most described and used EGM criteria for successful manifest AP ablation, such as fused atrio-ventricular (AV) signal, time from local ventricular EGM to the surface delta-wave, presence of Kent bundle potential, unipolar signal morphology were measured offline after each procedure.

Continuous atrio-ventricular electrical activity was defined as isoelectric interval of <5 msec between ventricular and atrial EGM (2). Time from local ventricular EGM to the surface delta-wave (in milliseconds) was measured from onset of the local ventricular EGM on the distal ablation probe to onset of the surface delta wave measured in any lead (10). Kent bundle potential was defined as a sharp and discrete deflection between local atrial and ventricular activation, preceding QRS complex onset during pre-excited sinus beats (10).

The unipolar pattern was categorized as having a QS morphology when the entire EGM displayed negativity. Conversely, if an initial positive deflection was present, the morphology was designated as rS (11).

Electrograms onset was defined as the first deflection from baseline with a slope greater than 45° at a sweep speed of 100 mm/sec (7).

### 2.6 Follow-up

Routine follow-up comprising anamnesis, clinical examination, 12-lead electrocardiography, 24-/48-h ECG-Holter recording, cardiac implantable electronic devices (CIEDs), interrogation was performed at 1, 3, 6, and 12 months and every 6 months later. In case of symptoms attributable to arrhythmic recurrence, each patient was invited to perform a Holter-ECG with a cardiological check-up or to visit the closest emergency department.

### 2.7 Statistical analysis

Continuous variables are expressed as median and interquartile range (25th-75th percentile), whereas categorical variables are presented as absolute frequencies and percentages.

## 3 Results

Baseline characteristics of the study population are summarized in Table 1. The median age was 26.5 (IQR 17- 45) years and 69% of the patients were males. Among the population 46 (92%) presented with overt atrio-ventricular pre-excitation. Distribution of the location of the different APs is shown in Table 2 with the most frequent AP location being in the right postero-septal region (28%, *n* = 14). For left-sided APs trans-septal puncture was performed in 11 (22%) cases whereas retro-aortic approach was the choice for 4 (8%) cases. A patent foramen ovale (PFO) was present in 4 patients (8%). Atrial activation map was obtained during sinus rhythm with ventricular pre-excitation, during antidromic atrioventricular tachycardia or coronary sinus pacing. Induction of tachycardia was achieved in 24 (48%) patients.

**Table 1:** Baseline characteristics are expressed as number and relative percentages or median and interquartile range.

Variables	*n* (%); Median (IQR)
Age	26.5 (17,45)
Male	31 (69)
Arterial hypertension	3 (6.7)
Diabetes	1 (2.2)
Concealed AP	3 (6.7)
Preserved LVEF	50 (100)

AP, accessory pathway; LVEF, left ventricular ejection fraction.

**Table 2:** Accessory pathway locations are expressed as number and relative percentages.

AP location	*n* (%)
Right postero-septal	14 (28%)
Left lateral	8 (16%)
Antero septal parahisian	10 (20%)
Left posterior	2 (4%)
Left postero lateral	4 (8%)
Right lateral	2 (4%)
Antero right	1 (2%)
Postero septal	2 (4%)
Right antero lateral	1 (2%)
Left postero septal	3 (6%)
Right antero lateral	1 (2%)
Left antero lateral	2 (4%)

The acute success rate was 100% and after a median follow-up of 10 (IQR 6–13) months neither reappearance of delta wave at the surface ECG nor recurrence of AVRT were ever recorded. Regarding ablation parameters, the median time to effect (TTE) was 2.0 (IQR 1.2–3.5) sec and for 34 (68%) patients the total ablation time was ≤60 s.

**Figure 2:**
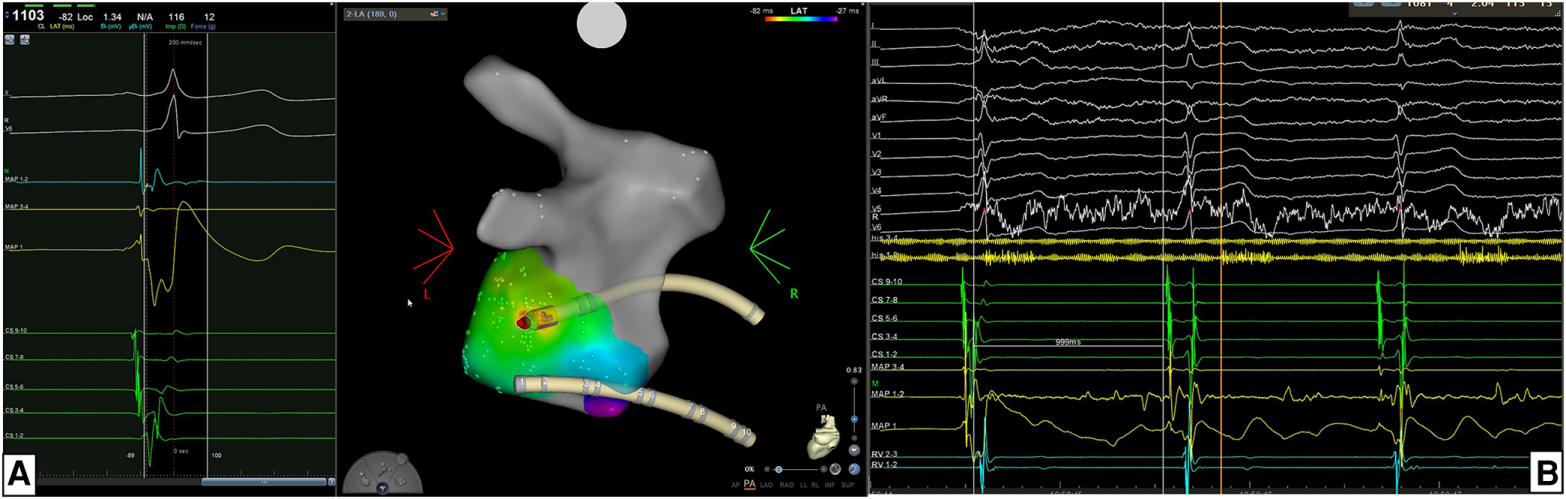
**(A)** Example of the location of accessory pathway (AP) being in the left postero-lateral region. For mapping and ablation of this left-sided AP an retro-aortic approach was performed. Atrial activation map was obtained during sinus rhythm with ventricular pre-excitation and a unipolar EGM with qS morphology and a continuous A-V EGM were recorded at the ablation point automatically marked by the system. **(B)** The acute success was 100% acutely with time to effect (TTE) was 2.0 s. In this case the AP block was achieved with just a single RF application.

In 50 (100%) patients AP block was achieved with just a single RF application (Figure 2). For these patients the target area was ≤0.1 cm^2^. As shown in Table 3 the overall median procedural time was 61 (IQR 57–70) minutes and for 37 (74%) patients no fluoroscopy was used while the overall median fluoroscopy time was 0.0 (IQR 0.0–0.4) min. The overall median mapping time was 13.1 (IQR 11.5–15.7) min with a median of 145 (IQR 111–200) mapping points per each map.

**Table 3:** Ablation characteristics and follow-up are expressed as number and relative percentages or median and interquartile range.

Procedural characteristics	*n* (%); Median (IQR)
Procedure time (min)	61 (57–70)
Fluoroscopy time (min)	0 (0–0.35)
Mapping time (min)	13 (11–15)
Mapping points	145 (111–200)
Continuous atrio-ventricular EGM	50/50 (100)
Delta-V interval (msec)	−10.0 (12.0–0.0)
Presence of AP potential	50 (100)
Presence of qS in unipolar	50/50 (100)
TTE (sec)	2.0 (1.2,3.5)
Total ablation time (sec)	60 (60,70)
Number of RF applications per AP	1 (1,1)
Acute success rate	50/50 (100)
Follow-up (months)	10 (6–13)
Recurrence of AP conduction during FU	0/50 (0)

EGM, electrogram; AP, accessory pathway; TTE, time-to-effect; RF, radiofrequency.

The median atrial signal amplitude was 0,57 (IQR 0.42–0.79) mV while the median delta-Ventricular interval was—10.0 msec (IQR −12.0–0.0), being less than—10 msec in 26 (52%) patients and ≤0 msec in all the cases.

The AP potential was visually found offline and its location corresponded to the optimal ablation point indicated by the proposed workflow in 50 (100%) patients.

Overall, no procedural and follow-up complications were recorded.

## 4 Discussion

Our study describes how the Delta Wave Automatic Mapping workflow enables precise automatic localization of the maximal pre-excitation of the accessory pathway in the absence of multipolar catheters. The WOI is set based of surface ECG leads to exclude the atrial signal. This, in synergy with specific automated annotation algorithms integrated in the CARTO Prime^TM^ software of the CARTO® 3 system, version 7 (Biosense Webster), defined the ablation target limiting the number and time of applications.

Conventionally, AP ablation success requires multiple RF pulses before conduction block sets in (12). The greater the number of ablation attempts, the higher the resulting tissue edema which diminishes the delivery of energy to the surrounding tissues, thereby reducing the likelihood of permanently ablating the AP. In our study, 50 (100%) out of 50 patients experienced disappearance of the ventricular pre-excitation after a single ablation delivered in the site automatically tagged with a median TTE of 2.0 (IQR 1.2–3.5) seconds.

Delta Wave Automatic Mapping algorithm objectively assisted the electrophysiologist in real-time identification of the accessory pathway location by automatically annotating the maximum negative slope of the unipolar ECG within the WOI previously defined in the bipolar EGMs and thus detecting the Kent signal, which consist in the earliest ventricular activation site.

Historically, the stability of EGM before ablation, the presence of Kent potential or atrioventricular signal continuity, a delta-wave to ventricle interval <0 msec, and the qS morphology on unipolar EGM, have been widely used as predictive parameters of successful ablation.

In our study we show that there is good correspondace between the AP location found with the automatic delta wave mapping and the traditional predictive parameters of success.

More in detail, a ventricular QS unipolar pattern with unipolar atrio-ventricular fusion (which was related with a success rate of 97% in Haissaguerre et al. 13) was present in all our cases matching the point automatically tagged by the system.

Even when considering the delta-wave to ventricle interval our method helped in localizing the area having a signal with a ventricular deflection that anticipates the delta wave. In our series of procedures, in the point automatically identified by the system, “delta-ventricular” median interval showed to be—10.0 msec (IQR −12.0–0.0) being less than—10 msec in 26 (52%) patients and ≤0 msec in all the cases.

Continuous electrical activity (defined as isoelectric interval of <5 msec between ventricular and atrial electrograms) (2) has been considered another criteria for successful AP ablation given the anatomical continuity of myocardial tissue between atria and ventricles provided by the Kent bundle. In our study, for each case we were able to find continuous electrical activity in the offline analysis of the signals and this type of EGM corresponded to the ablation target point automatically identified by the system. This is again due to the ability of the proposed workflow to identify the maximum negative slope of the unipolar EGM, corresponding to the Kent bundle.

In our study the ablation and mapping catheter used was the ST or STSF. Currently, novel ablation catheters such as the QDOT micro catheter (Biosense Webster) have emerged with microelectrodes potentially capable of enhancing the mapping resolution. It is not possible to apply our proposed algorithm considering the signals coming from the microelectrodes because the CARTO system does not perform time annotation on these µ-signal. However, once the delta wave automatic mapping has been applied considering the conventional electrodes, the QDOT catheter could be positioned in the area identified by the algorithm ad microelectrode signals could be qualitatevily evaluated by the operator to further confirm the optimal ablation location (14).

In our study, the median procedural time averaged 61 (IQR 57–70) min, significantly shorter than the findings reported by Mykazaki et al. (15), who studied 17 APs with 6 manifest bypass tracts, indicating a median procedural duration of 90 (IQR 72.5–110) min, as well as by Fernandez and colleagues in their study involving 35 patients, 19 with manifest AP, where the procedural duration was 129.7 ± 9.1 min.

All patients obtained successful ablative end points of the accessory pathways.

While the majority of older reports indicate that EA mapping methods did not demonstrate a superior success rate compared to the traditional fluoroscopy-guided approach (16–18), a recent study by Bergonti et al. suggests potential long-term benefits associated with the use of an EA mapping system (18).

Moreover, ablation with near-zero x-ray approach reduced exposure to fluoroscopy ensuring safety and procedural efficacy (19) with a significant decrease in estimated risks of cancer incidence and mortality (20). In our scenario, we adopted the near-0 fluoroscopy technique [overall median fluoroscopy exposure of 0.0 (IQR 0.0–0.4) min].

Therefore, despite the limited data available for comparison, the delta-wave automatic workflow shows potential in reducing the overall procedure duration by aiding in precise identification of the AP location, thereby decreasing ablation times.

However, it is important to underline that next to the automatic method proposed in this article it is always important to leverage all the knowledge and expertise the operator has gained and to combine it with the modern EAM techniques to achieve the best results. Conventional mapping approaches have demonstrate over years to be highly effective and the lessons learned from complicated cases mapped with a conventional point-by-point approach should be always kept in mind when integrating EAM and automatic mapping algorithms.

## 5 Limitations

Despite the innovations brought by our automatic workflow in the field of AP mapping and ablation, our study has some limitations that should be pointed out.

The study population consisting only in a group of patients with stable or intermittent ventricular pre-excitation. In our study we did not enroll re-do ablation procedures and did not compare the efficacy of the automatic mapping workflow with the standard 3D electroanatomic mapping strategy.

## 6 Conclusions

Overall, Delta Wave Automatic Mapping is the integration of a conventional WOI-modifying automatic mapping technique and represents a significant advance in the accurate localization of maximum ventricular preexcitation in manifest accessory pathways. This approach leverages the power of technology and algorithmic sophistication to improve the efficiency and accuracy of APs localization. We believe that delta wave automatic mapping combined with conventional approaches could help in improving the efficiency in manifest AP localization and ablation.

## Data Availability

The raw data supporting the conclusions of this article will be made available by the authors, without undue reservation.

## Aknowledgments

We would like to thank J. Janjic, C. Di Martino, R. Martino, M. Fioravanti and M. Giulianini from Biosense Webster for their technical support.
